# Effects of the Tyler Twist Technique Versus Active Release Technique on Pain and Grip Strength in Patients With Lateral Epicondylitis

**DOI:** 10.7759/cureus.46799

**Published:** 2023-10-10

**Authors:** Fatimah Kazi, Deepali S Patil

**Affiliations:** 1 Physiotherapy, Ravi Nair Physiotherapy College, Datta Meghe Institute of Higher Education and Research, Wardha, IND

**Keywords:** lateral epicondylitis, tennis elbow, active release technique, tyler twist technique, eccentric exercise

## Abstract

Background

Lateral epicondylitis is one of the most prevalent repetitive strain injuries or overuse injuries of the upper limb. Lateral epicondylitis also known as tennis elbow can be caused by repeated wrist and forearm movements. Treatment strategies have evolved significantly to treat tennis elbow, ranging from simple exercises to the use of various electrotherapy modalities. Soft-tissue release treatments such as myofascial release and active release techniques (ARTs) have also been tested. Better therapeutic approaches for chronic lateral epicondylitis remained a point of contention until recently when additional therapy alternatives became available. The purpose of this study was to investigate and assess the physiotherapy alternatives for lateral epicondylitis.

Methods

We did a comparative study between the Tyler twist technique and the ART in patients suffering from lateral epicondylitis. This study included 30 individuals based on inclusion and exclusion criteria. Group A patients were taught the Tyler twist technique exercise along with conventional therapy. Patients in group B were treated with ART and conventional therapy. The treatment session lasted for 30 minutes including appropriate breaks in between the session. Outcome measures for this study were a numerical pain rating scale (NPRS) and grip strength measurement by a handheld dynamometer. The unit of measurement of grip strength was kilograms (Kg). Descriptive and inferential statistics were used in the statistical analysis.

Results

A total of 30 subjects with lateral epicondylitis were included. Participants were randomly distributed into two groups, that is, 15 in each group. Group A was the Tyler twist technique group and group B was the ART group. The treatment was given in four sessions each week for three weeks. The pain reduced from 5.8 to 2 after the Tyler twist technique in group A and 5.53 to 3.46 after the ART in group B. On comparative analysis, the post-treatment mean grip strength of the Tyler twist technique group was 24.13 kg and that of the ART group was 21.33 kg. The p-value was statistically significant with a value of 0.0001. The Tyler twist technique was more effective in improving the grip strength than ART.

Conclusion

The Tyler twist technique was found to be a more effective therapeutic intervention for lateral epicondylitis as a significant decrease in pain on the NPRS and an increase in grip strength on a handheld dynamometer were observed.

## Introduction

Lateral epicondylitis, popularly acknowledged as "tennis elbow," is one of the most prevalent repetitive strain injuries of the upper limb [1]. There are many causative factors of this condition, with playing tennis accounting only for 5% of lateral epicondylitis cases [2]. The occupations, sports activities, and activities at home in which extending the wrist is required leads to this condition. It is a common condition springing up due to overuse of the elbow joint. It regularly and frequently involves the extensor muscle tissue of the forearm and causes inflammation around the lateral part of the humerus, therefore, called lateral epicondylitis.

For instance, there are occupations in which there are continuous repetitive movements like plumbing; playing a musical instrument like guitar or violin; painting, weaving; the usage of screwdrivers, slicing meat and cutting vegetables; turning doorknobs; and other twisting movements. Picking up hefty gadgets with an extended wrist also aggravates the pain. A strong relationship exists between occupation and industry work in developing tennis elbow [3]. It is a repetitive strain injury of the extensor muscles of the forearm. Anatomically the muscle involved is the extensor carpi radialis brevis (ECRB). It originates from the humerus's lateral epicondyle and escapes with extensor carpi radialis longus. Extensor carpi radialis longus integrates further into the bottom of the second metacarpal, while ECRB attaches to the base of the third metacarpal [4]. It is a strong extensor of the hand.

The pathology of lateral epicondylitis is defined as continual degeneration of the tendons of extensor muscle mass joined at the lateral aspect of the epicondyle of the humerus, additionally referred to as tendinosis. When the excessive use of the extensor muscles of the wrist exceeds the tendon's resistance, a micro-tear emerges, and the accumulation of those micro-tears finally leads to tendinosis [5]. Symptoms of tennis elbow include painful gripping activity, specifically rotation or extension of the wrist using of hack-shaw, plier, screw, and penknife hand drill. Predominant symptoms of this condition include lateral elbow pain on gripping or resisted wrist dorsiflexion [6]. Tyler twist technique is an eccentric exercise (EE) that involves elongation of the musculotendinous unit while a load is applied to it. Little was known regarding the effects of eccentric workouts, which result in decreased pain in people suffering from tendinopathy. EE has proved to be a beneficial treatment for several types of tendinopathies, including calf muscle tendinopathy and patellar tendonitis. The tendon is exposed to higher stresses in EEs than in concentric exercises, resulting in a stronger remodeling stimulus. In EEs, the musculotendinous unit is also lengthened hence lesser strain is placed on the tendon while performing a movement [7].

Active release technique (ART) is a soft tissue movement-based technique. It was developed by Dr PM Leahy. In this method, the therapist makes use of profound digital tension over the area of pain and instructs the subject to move the tissue from a shorter position to a lengthened position [8].

## Materials and methods

This study aimed to evaluate the efficiency of the Tyler twist technique versus the ART on pain and grip strength in patients with lateral epicondylitis. The study began after receiving approval from the Institutional Ethics Committee (IEC). It was an experimental comparative study, and the type of study was interventional. The duration of this research was six months. The total number of participants was 30, which is 15 in each group. Two groups were formed, group A was the Tyler twist technique group and group B was the ART group. Simple random sampling using the envelope method was done to distribute the patients into the two groups.

The inclusion criteria were males and females aged between 20 and 65 years, patients with right-hand dominance as well as left-hand dominance, who were diagnosed with lateral epicondylitis, and those who had tested positive for Cozen’s test, Mill’s test, Maudsley’s test, or all of the three. Exclusion criteria included participants who had any recent traumatic injury around the elbow joint, wrist, fingers, and shoulder. Participants were informed about the study, and consent was obtained.

Pre- and post-interventional assessment was carried out, using the numerical pain rating scale (NPRS) and measurement of grip strength. Pain assessment was done by showing and explaining to the patient the NPRS and then asking them to rate their pain on the scale according to their level of discomfort. Grip strength was assessed using a handheld Jamar dynamometer. The patient was seated on the chair with the affected hand to be tested resting on the armrest of the chair. The patient's elbow rested on the armrest at an angle of 90°. The wrist was over the arm of the chair, with the thumb pointing up. Then the patient held the dynamometer with the fingers and thumb around its handle. To measure the strength, he squeezed the dynamometer as much as he could. The unit of measurement of grip strength was kg.

Treatment was given four times a week for three weeks. All the participants completed the exercise regimen. Statistical analysis was performed using descriptive and inferential statistics, including the chi-square test, Student's paired and unpaired t-tests, and SPSS 27.0 software (IBM Corp., Armonk, NY, USA), with p<0.05 recognized as the level of significance. The results of the statistical analysis are shown in Table 1, Table 2, and Table 3. The regimen for both groups is described below.

**Table 1:** Comparison of grip strength in active release technique and Tyler twist technique at pre- and post-treatment ART, active release technique

Technique	Pre t/t	Post t/t	Mean difference	Student’s paired t-test t-value
ART	19.06±4.84	21.33±4.65	2.26±0.45	19.17 P=0.0001,S
Tyler twist technique	19.66±4.40	24.13±5.54	4.46±1.45	11.87 P=0.0001,S
Comparison between two techniques (Student’s unpaired t-test)				
t-value	0.35 P=0.72, NS	1.49 P=0.45, NS		

**Table 2:** Distribution of patients according to their age in the two techniques ART, active release technique

Age group (yrs)	ART	Tyler twist technique	ϗ2-value
21-30 yrs	4(26.67%)	5(33.33%)	1.25 P=0.86, NS
31-40 yrs	3(20%)	3(20%)	
41-50 yrs	3(20%)	4(26.67%)	
51-60 yrs	3(20%)	1(6.67%)	
>60 yrs	2(13.33%)	2(13.33%)	
Total	15(100%)	15(100%)	
Mean±SD	42.73±14.30	40.20±14.20	
Range	23-65 yrs	22-65 yrs	

**Table 3:** Comparison of NPRS score in active release technique and Tyler twist technique at pre- and post-treatment ART, active release technique

Technique	Pre t/t	Post t/t	Mean difference	Student’s paired t-test t-value
ART	5.53±1.12	3.46±1.06	2.06±0.45	17.48 P=0.0001, S
Tyler twist technique	5.80±0.94	2.00±0.84	3.80±0.86	17.07 P=0.0001, S
Comparison between two technique (Student’s unpaired t-test)				
t-value	0.70 P=0.48, NS	4.19 P=0.0001, S		

In group A, EEs were based on the principle of strengthening the muscle. These exercises improve strength and decrease pain. Increasing the weight stresses the tendon and serves as the foundation for the strengthening regime. These exercises were specifically designed to benefit the injured tendon. It has been studied that excellent outcomes with eccentric training were due to the effects on neovascularization [9]. The Tyler twist technique was taught to the patient using a FlexBar®. The FlexBar® exercise sequence (also known as “The Tyler twist”) is done as follows: 1. Hold yellow-colored (starting with the least resistance) FlexBar® in the involved hand with a maximally extended wrist. 2. Grab another end of FlexBar® with an uninvolved wrist. 3. FlexBar® is twisted with an uninvolved wrist while keeping the affected wrist extended. 4. Bring your arms in front of you, elbows extended at the same time maintain the twist in FlexBar® with the uninvolved wrist in maximum flexion and the affected wrist in maximum extension. 5. Now permit the FlexBar® to "untwist" by letting the involved wrist eccentrically contract by flexing the wrist. The FlexBar® exercise was completed three times for a total of 15 repetitions. Every repetition takes four seconds, so every set of 15 repetitions was separated by a 30-second pause. Patients proceeded toward the other level color FlexBar® after completing three sets of 15 for three weeks, indicating a higher level of eccentric resistance [10]. Conventional physiotherapy consists of ultrasound therapy. Five to eight minutes of pulsed ultrasound treatment at the tenoperiosteal joint of the ECRB and at the area of tenderness with a 1:4 pulse ratio of 1 MHz at 1.5 W/cm^2^ was done. The total treatment session lasted up to 20-30 minutes.

In group B, ART was given when the patient was seated comfortably, with the elbow in flexion while relaxing on the treatment couch, the wrist in a neutral position, and the forearm in a mid-prone posture. Then putting stress on muscles distal to their attachment, the physiotherapist focused on the extensor carpi radialis longus and brevis muscles. The starting position was with the elbow bent and the wrist in a normal position. While the therapist was holding and applying the pressure on the muscles with her fingers/thumb, the patient was instructed to extend the elbow, and the wrist should be flexed and pronated. Meanwhile, the pressure was maintained in the proximal direction in an attempt to break up adhesions.

**Figure 1:**
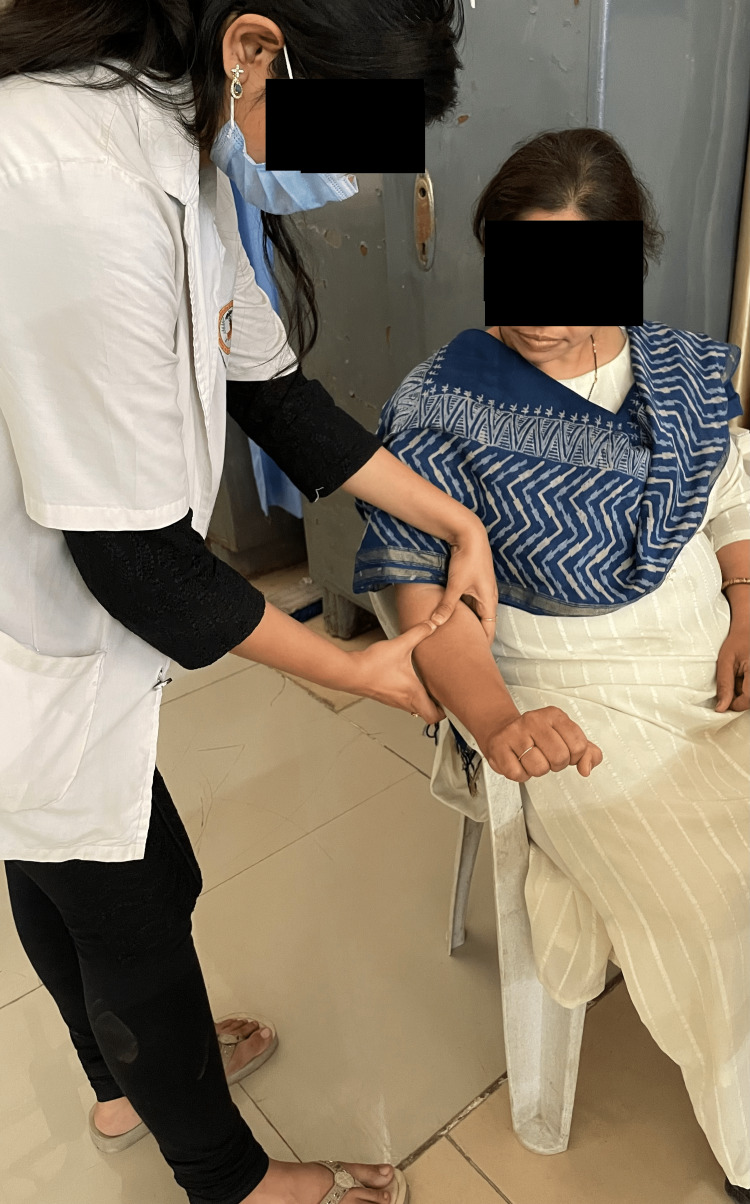
Therapist performs ART ART, active release technique

**Figure 2:**
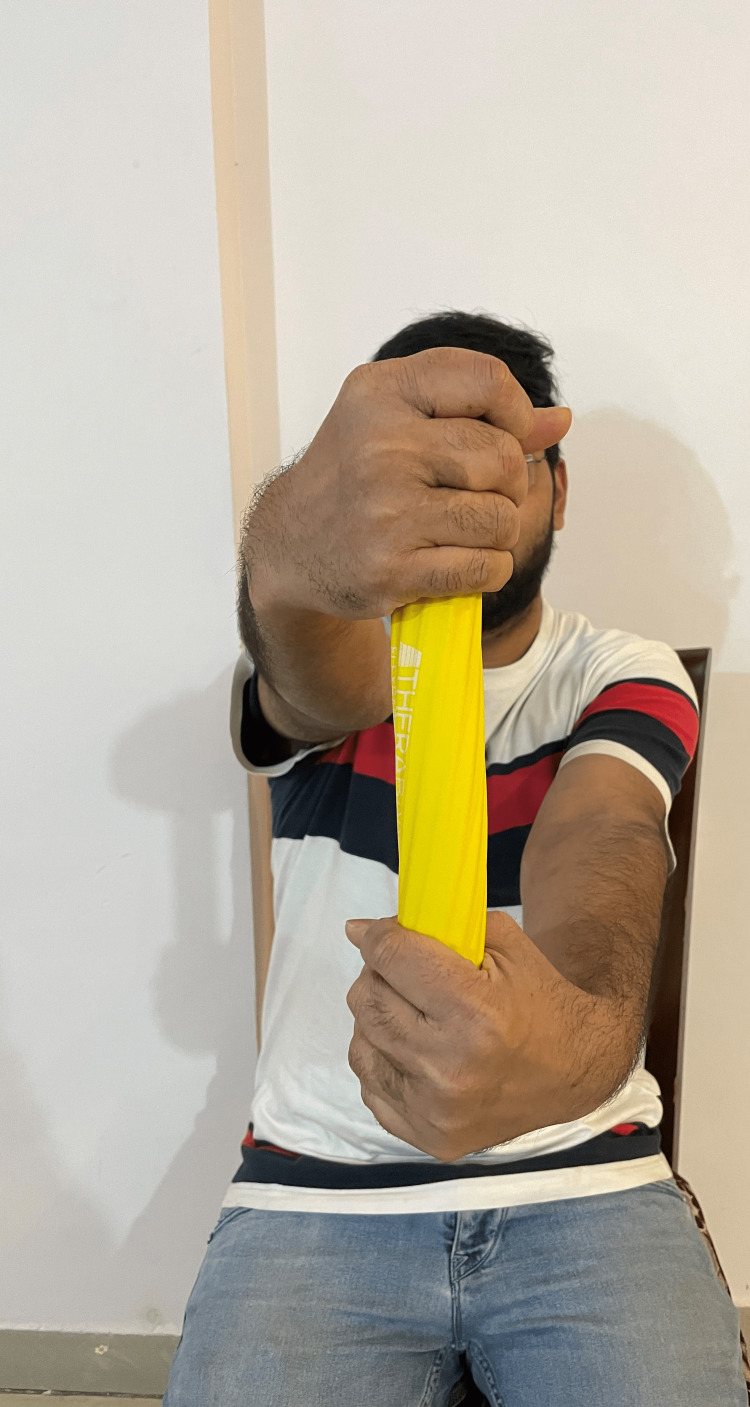
Patient performs Tyler twist technique using a yellow Thera-Band Flexbar. It is the starting position of the technique

**Figure 3:**
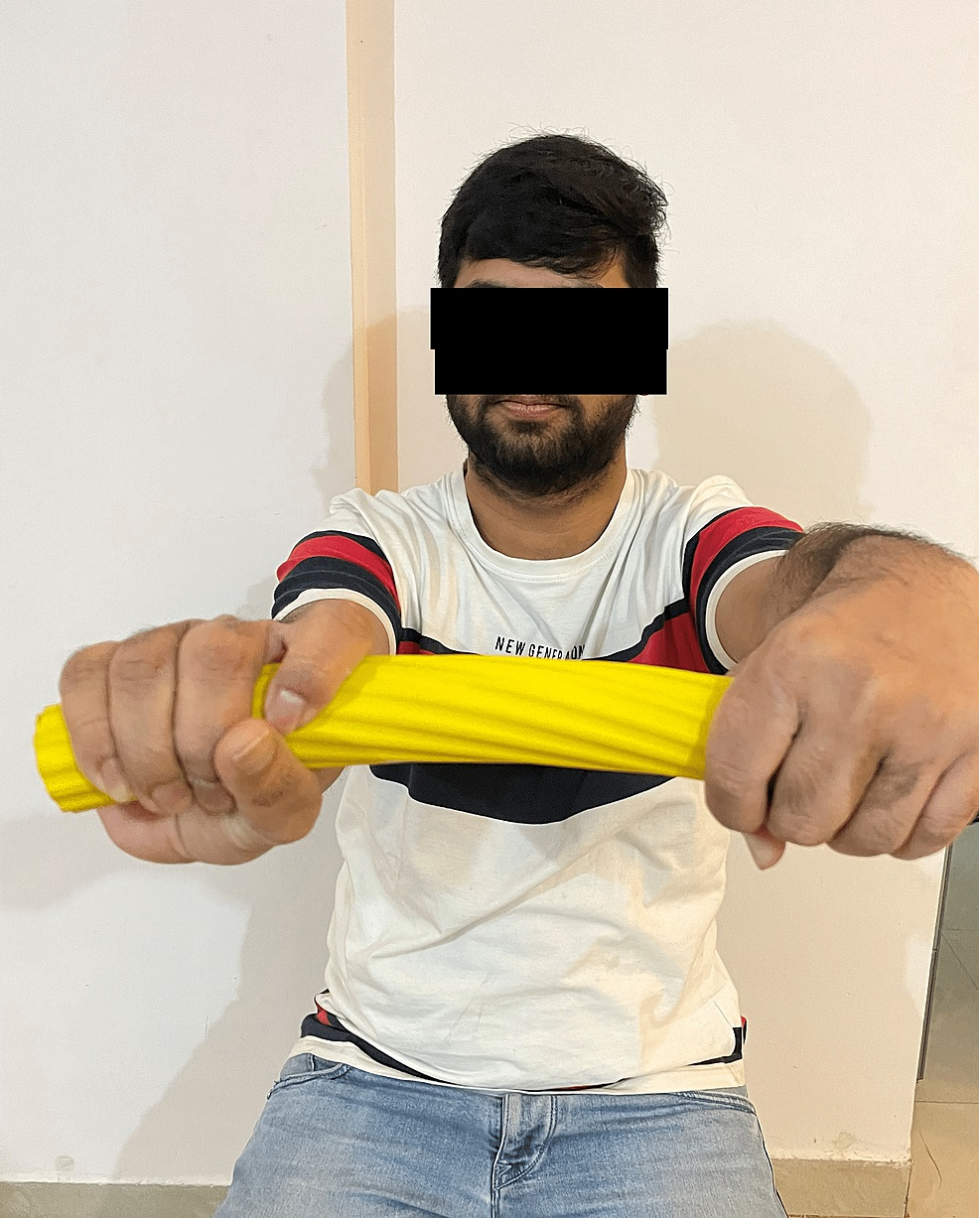
End position of Tyler twist technique

ART was performed three times per week for up to three weeks, with a maximum of 15 repetitions for 10 minutes. Conventional physiotherapy consists of ultrasound therapy. A total of 5-8 minutes of pulsed ultrasound treatment at the tenoperiosteal joint of the ECRB and the area of tenderness with a 1:4 pulse ratio of 1 MHz at 1.5 W/cm^2^ was done. The total treatment session lasted up to 20-30 minutes. The therapist delivers the ART to the patient as shown in Figure 1. The patient performs the Tyler twist technique in Figure 2 and Figure 3.

## Results

**Figure 4:**
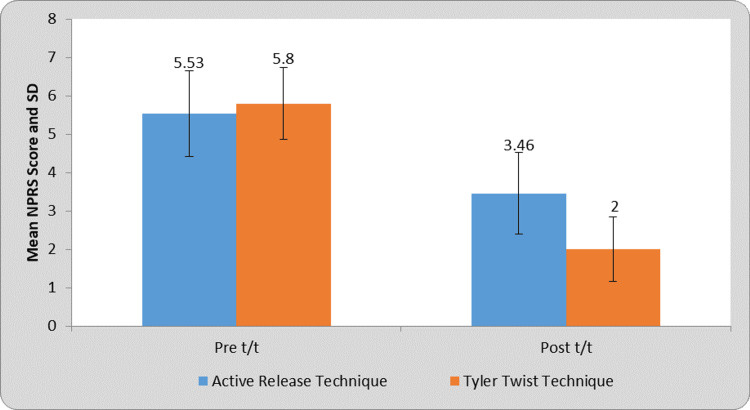
Pre- and post-treatment comparison of NPRS score of ART group and Tyler twist technique group ART, active release technique

Figure 4 shows a comparison of NPRS score in ART and Tyler twist technique at pre- and post-treatment. The pain was reduced from 5.8 to 2 after the Tyler twist technique in group A and 5.53 to 3.46 after the ART in group B. Group A showed much reduction in pain than group B on NPRS score.

**Figure 5:**
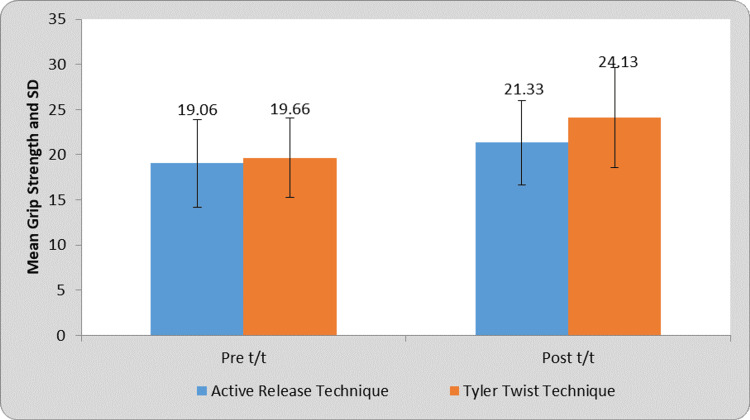
Pre- and post-treatment comparison of grip strength in ART and Tyler twist technique ART, active release technique

Figure 5 shows a comparison of grip strength in the art and Tyler twist technique at pre- and post-treatment evaluation. The Tyler twist technique group was more effective by 24.13 in improving the grip strength than the ART group, which was at 21.33.

According to the results obtained, it is clear that the Tyler twist technique group showed a significant reduction in pain and an increase in grip strength than the ART group.

## Discussion

The goal of this research was to assess the efficiency and perform a comparison between the Tyler twist technique and the ART in patients with lateral epicondylitis. We found out in this study that females were more affected than males, 93.33% and 73.33% in groups A and B, respectively. The dominant hand, which was the right hand (86.67%), was more affected than the non-dominant one. Tyler twist technique group showed a significant improvement in the grip strength, that is, an increase from an average of 19.66 kg to 24.13 kg.

Decreased functionality and decreased grip strength are frequently impacted due to pain in the tennis elbow. In lateral epicondylitis, grip strength is a significant aspect used to measure the severity of loss of strength [11]. Load reduction on the tendon is a successful prevention technique that must be accompanied by the development of tissue resilience to allow for progression toward the desired load by training the mechanical characteristics of the tendon. An effective strategy to change the load is to have the patient work below their pain threshold and undertake activities that load the tendon below the level of exacerbated discomfort [12]. However, a study showed that EE at higher intensity was shown to be efficient in increasing muscle mass and muscle strength as assessed by improvement in muscle girth. Subgroup analyses in that study implied that the advantage of EE to improve muscular strength and bulk appears to be linked to the greater loads produced during eccentric contractions. In addition, greater forces produced during this form of exercise may facilitate eccentric training's dominance in terms of increasing strength and muscle mass [13]. 

EEs specifically target the eccentric phase of muscle contractions. They work by placing greater emphasis on the controlled lengthening of a muscle under tension/load. In a study, it was found that after 26 weeks, a 12-week isolated eccentric rotator cuff exercise plan is useful for shoulder function and discomfort in individuals with rotator cuff tendinopathy. According to the findings, carrying out two EEs twice a day is as helpful as doing six concentric/eccentric exercises per day in individuals with rotator cuff tendinopathy [14]. This case study bears a resemblance to our research in revealing a congruent pattern of results. Specifically, both researches converge on the observation that the strategic integration of EE modalities is associated with a substantial augmentation of muscular strength. It was observed in another pertinent case report that patients with lateral epicondylitis reacted satisfactorily with ART as well as evidenced by decreased discomfort and improved functional results. The patient underwent a total of five sessions of ART over the course of three weeks performed on the soft tissue of the elbow [15].

The imitation of this study is that an increase in the bulk of the extensor muscles was not measured after progressively loading the muscle. The number of weeks required for treatment could have been extended. Long-term follow-up of the patient is also necessary but was not possible in the present study. The lacunae of our study was that the pressure provided by the therapist, while important, was not standardized and might vary across people. We used no apparatus to measure the applied pressure. This study's findings revealed that the EEs (Tyler twist technique) is better than ART to reduce pain and improve grip strength. 

## Conclusions

To conclude, our study has demonstrated that the Tyler twist technique exhibits superior effectiveness when compared to the ART. On the NPRS, the pain was reduced from 5.8 to 2 after the Tyler twist technique in group A and 5.53 to 3.46 after the ART in group B. The post-treatment grip strength of the Tyler twist technique group was more effective by 24.13 kg in improving the grip strength than the ART group, which was at 21.33 kg.

In conclusion, our comparative study yielded insightful findings. While both therapies reduced pain almost similarly, a significant difference in their influence on grip strength appeared. Our research unambiguously shows that the Tyler twist approach outperforms ART in terms of dramatically increasing grip strength. This discovery not only emphasizes the Tyler twist technique's practical benefits for individuals looking to improve their grip strength, but it also highlights its special value in specific clinical or performance-oriented circumstances. Future studies may look at these approaches in greater depth and with bigger sample sizes. Nonetheless, our findings give significant insight to practitioners and people trying to pick the most effective intervention for their unique goals, particularly in situations where grip strength gain is critical.
